# Wavelet Transform Fuzzy Algorithms for Dermoscopic Image Segmentation

**DOI:** 10.1155/2012/578721

**Published:** 2012-04-11

**Authors:** Heydy Castillejos, Volodymyr Ponomaryov, Luis Nino-de-Rivera, Victor Golikov

**Affiliations:** ^1^National Polytechnic Institute 04430, Mexico city, DF, Mexico; ^2^UNACAR, 24180 Ciudad del Carmen, CaM, Mexico

## Abstract

This paper presents a novel approach to segmentation of dermoscopic images based on wavelet transform where the approximation coefficients have been shown to be efficient in segmentation. The three novel frameworks proposed in this paper, W-FCM, W-CPSFCM, and WK-Means, have been employed in segmentation using ROC curve analysis to demonstrate sufficiently good results. The novel W-CPSFCM algorithm permits the detection of a number of clusters in automatic mode without the intervention of a specialist.

## 1. Introduction

According to the World Health Organization, skin cancer is the most common form of human cancer. It is estimated that over one million new cases occur annually. Additionally, the diagnosis of skin cancer is particularly important because melanoma can be cured with a simple excision if detected early.

The term “skin cancer” refers to three different conditions that are listed below in ascending order of mortality:

- basal cell carcinoma (or basal cell *carcinoma epithelioma*),
- squamous cell carcinoma (the first stage of which is called *actinic keratosis*),
- * melanoma*.

Melanoma is generally the most serious form of skin cancer because it tends to spread (metastasize) throughout the body quickly.

To diagnosis skin cancer, doctors usually remove all or a part of the growth by performing a biopsy, but this is considered an invasive technique. Alternatively, a technique called dermatoscopy reduces the need for a biopsy by utilizing a dermatoscope. Dermatoscopy is a particularly helpful standard method of diagnosing the malignancy of skin lesions [1]. One of the major advantages of dermatoscopy is an increase in accuracy compared with naked-eye examination (up to 20% in the case of sensitivity and up to 10% in the case of specificity), thereby reducing the frequency of unnecessary surgical excisions of benign lesions [2–4].

In addition, several instruments designed for a computer-aided diagnosis (CAD) of skin lesions have been proposed. These usually work in four steps: data acquisition of skin (dermoscopic images), segmentation, feature extraction, and classification. The most relevant step is the segmentation process because it provides fundamental information to the next stages. Image segmentation is the process of adequately grouping pixels into a few regions, where pixels within a group share some similar characteristics. Automated analysis of the edges, colors, and shape of the lesion relies upon an accurate segmentation, and this is an important first step in any CAD system. However, irregular shape, nonuniform color, and ambiguous structures make the problem difficult.

Image segmentation can be classified into three categories (a) *Supervised*: these methods require the intervention of the analyst, who should specify the sections of skin or lesions within the image [4, 5]. (b) *Automatic*: also known as unsupervised methods, these systems attempt to find the lesion borders without any intervention from an analyst. (c) *Semiautomatic*: this describes a combination of manual and automatic segmentation.

Many segmentation methods have been developed for border detection in pigmented skin images, and they traditionally focus on dermoscopic images.

In [6], automatic adaptive thresholding (AT) has been proposed where the main idea is to segment an image comparing the color of each pixel with a threshold. In [7], the authors use a variant of a region growing and merging technique called statistical region merging (SRM) to segment an image. The SRM technique has been proven to be robust for the segmentation of color images, improving the detection rate of skin lesions.

A recent review of methods for segmentation of skin lesions in dermoscopic images [5] suggests that clustering is the most popular segmentation technique, most likely due to its robustness.

However, in some cases, it can be difficult to perform good segmentation because of hair occlusions within the pigmented skin lesion. In this case, preprocessing methods, such as those proposed in the following papers [8, 9], should be employed before segmentation.

Texture is an important characteristic of image analysis for both natural scenes and medical images. The *wavelet transform *(WT) provides an ideal representation of texture analysis presenting spatial-frequency properties via a pyramid of tree structures that is similar to subband decomposition. The hierarchical decomposition enables an analysis of the high frequencies in the image, which is important for the segmentation process. 

Several techniques use the image features within a WT domain during the segmentation process. In Bello [10], image data are first decomposed into channels for a selected set of resolution levels using wavelet packets transform. Then, Markov random field (MRF) segmentation is applied to the subband coefficients for each scale, starting with the coarsest level and propagating the segmentation process from the current level to segmentation at the next level. Strickland and Hahn [11] use the image features extracted in the WT domain for detection of microcalcifications in mammograms using a matching process and some *a priori *knowledge on the target objects. Zhang and Desai [12] employ a Bayes classifier on wavelet coefficients to determine an appropriate scale and threshold that can separate segmentation targets from other features.

In this paper, we propose an efficient approach for the segmentation of dermoscopic images based on a clustering process. Our novel approach uses feature extraction in wavelet transform space before proceeding to the segmentation process. The main difference with other algorithms presented in the literature is in the usage of information from three color channels (RGB space) in wavelet transform space gathering the color channels via a *nearest neighbor interpolation* (NNI).

The paper is organized as follows.  Section 2 presents a methodology; Section 3 exposes the proposed framework; Section 4 discusses the evaluation criteria applied; Section 5 contains the simulation results; Section 6 contains the contribution of this work; Section 6 concludes the paper.

## 2. Methodology

### 2.1. Statistical Region Merging

In [7], the authors use a variant of a region growing and merging technique called statistical region merging (SRM) to segment an image. The SRM technique has been proven to be robust for the segmentation of color images, improving the detection rate of skin lesions. This framework includes the following strategy.

First, a registered image *I* in RGB space is considered an observation of a true image I*, in which pixels are perfectly represented by a family of distributions from each of the observed color channels. The color channel values for every pixel are replaced by Q independent random variables with values from (o, g/Q), where the value Q represents the number of regions that should be generated.

**Figure 1:**
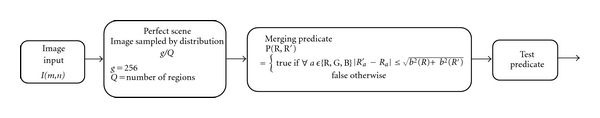
Block diagram of statistical region merging.

The predicate of regions is defined as

(1) $$\begin{array}{c} P\left(R,R^{\prime }\right)=\{\begin{array}{cc} \text{true} & \text{if}\;\forall a\in \left\{R,\;G,\;B\right\}\left|R_{a}^{\prime }-R_{a}\right|\\ & \;\;\;\;\;\leq \sqrt{b^{2}\left(R\right)+b^{2}\left(R^{\prime }\right)}\\ \text{false} & \text{otherwise}, \end{array} \end{array}$$

where *R* and *R*′ represent the two regions being tested and *R*_*a*_ denotes the observed average. *R* and *R*_|*p*|_ are the set of regions with *p* pixels. The SRM framework is shown in Figure 1. 

### 2.2. K-Means Clustering Algorithm

This algorithm is an unsupervised clustering algorithm that classifies the input data points into multiple classes based on their inherent distance from each other [13]. It works in an iterative manner according to the following steps.

1. Choose initial centroids *m*_1_,…, *m*_*k*_ of the clusters *C*_1_,…, *C*_*k*_.
2. Calculate new cluster membership. A feature vector *x*_*j*_is assigned to the cluster *C*_*i*_ if and only if
  (2) $$i=\underset{k=1,\ldots ,K}{\text{argmin}}{||x_{j}-m_{k}||}^{2}.\;\;$$
3. Recalculate the centroids for the clusters according to
  (3) $$m_{i}=\frac{1}{\left|C_{i}\right|}\underset{x_{j}\in C_{i}}{\sum }x_{j},\;$$
  where *x*_*j*_ belong to dataset *X* = {*x*_1_,…, *x*_*i*_ …, *x*_*N*_}.
4. If none of the cluster centroids were changed, finish the algorithm. Otherwise go to step 2.

### 2.3. Fuzzy C-Means Algorithm

The algorithm finds the center of “*n*” number of clusters iteratively by adjusting their position and evaluating an objective function. Additionally, it permits more flexibility by introducing partial membership to other clusters. The classical algorithm uses the following objective function:

(4) $$E=\overset{C}{\underset{j=1}{\sum }}\overset{N}{\underset{i=1}{\sum }}\mu_{ij}^{k}{||x_{i}-c_{j}||}^{2},$$

where *μ*_*ij*_^*k*^ is the fuzzy membership of the pixel *x*_*i*_, the cluster identified by its center *c*_*j*_, and *k* is a constant that defines the fuzziness of the resulting partitions.

 The membership value is proportional to the probability that a pixel belongs to some specific cluster where the probability is only dependent on the distance between the pixel and each independent cluster center. Consequently, the criterion *E* has a minimal value when the pixels are nearby the corresponding cluster center. Higher membership values are assigned to these nearby pixels while lower membership values are assigned to the pixels that are far from a center. This algorithm runs with the clusters number and initial center positions as previously determined. The algorithm then determines how many pixels belong to each cluster. The membership function and centers are determined as follows:

(5) $$\mu_{ij}=\frac{1}{\sum_{m=1}^{C}||x_{i}-c_{j}||/{||x_{i}-c_{m}||}^{2/\left(k-1\right)}},\;$$

(6) $$c_{i}=\frac{\sum_{j=1}^{N}u_{ij}^{k}x_{j}}{\sum_{j=1}^{N}u_{ij}^{k}}.$$

The FCM algorithm runs four simple steps.

1. The center is initialized with the first value “*t*” of the data to be equal to zero, and this value is used as a counter for the number of iterations.
2. The fuzzy partition membership functions *μ*_*ij*_ are initialized according to (5).
3. The value “*t* = *t* + 1” is changed and novel centers are computed using (6).
4. Steps 2 and 3 run until criterion *E* converges.

Criterion *E* approaches a minimum value when its variations are decreased according to the restriction that a user selects. The algorithm can also be interrupted if a user determines that only a certain number of iterations are required [13].

### 2.4. Cluster Preselection Fuzzy C-Means

The FCM algorithm is one of the most common procedures for image segmentation but has the following drawback: the number of clusters needs to be predetermined by a user. Therefore, the user may not select the correct number of clusters for a given specific application. Therefore, a method that uses fuzzy logic to find the number of clusters can reproducibly select the correct number of clusters. To achieve this, we take into consideration the difference between the max (*V*_max⁡_) and the min (*V*_min⁡_) values of intensity in an image *D* = *V*_max⁡_ − *V*_min⁡_. Using these proportions, the algorithm determines the optimal number of clusters. Specifically, image data are analyzed to determine the centers, thus reducing the operational time of the FCM algorithm. The first data classification for our fuzzy system is called “Distance” and has a total of six fuzzy sets, “minimum,” “shorter,” “short,” “regular,” “large,” and “maximum” (see \). The classification for our fuzzy system called “Size” has a total of five fuzzy sets, “Min,” “Small,” “Medium,” “Big,” and “Max” (see Table 2). Finally, the classification for our fuzzy system called “Cluster” has five fuzzy sets, “Very few,” “Few,” “Some,” “Many,” and “Too Many” (see Table 3).

Finally, the fuzzy system “cluster” contains five fuzzy sets that are applied in the determination of the centers using 30 fuzzy rules, reducing the operational time of the FCM algorithm. The overall response of the fuzzy system can be represented as follows:

(7) $$Q\left(c\right)=\underset{i}{\max}\left\{\min\left\{\min\left\{\mu_{\text{distancia}}\left(d_{0}\right),\mu_{\text{size}}\left(s_{0}\right)\right\}{,\mu }_{i}\left(d_{0},s_{0},c\right)\right\}\right\},$$

where *i* = 1, 2, …, 30 fuzzy rules, *distance is *{minimum, shorter, short, regular, large, maximum} and size is {min, small, medium, big, max}. 

**Figure 2:**
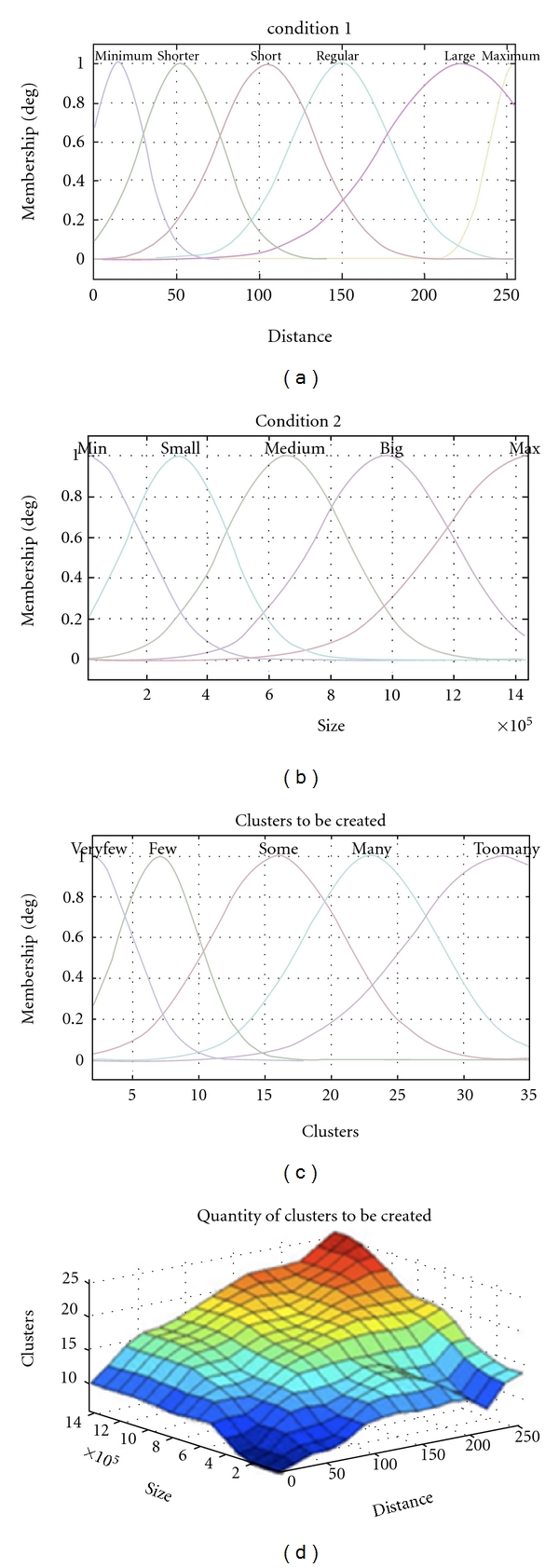
Preselection of the number of clusters.

In Figure 2 we can see the membership functions with a gaussian distribution of three conditions and the total number of clustrers.

In the second phase, the number of clusters and their centers are already known, simply requiring dividing the difference *D* into the “*N*” clusters to determine the centers:

(8) $$\begin{array}{c} \begin{array}{c} c_{j}=j\frac{D}{N}\;j=1,2,3,\ldots ,N, \end{array} \end{array}$$

where “*N*” represents the number of clusters to be created and “*j*” is a counter to define all the centers.

This looks like a hard type of algorithm, but the centers are still rather far from the final ones. Therefore, there are still a certain number of iterations that should be applied to find them, but the number of iterations is far fewer than for the original system, reducing the required computation time.

The RGB image is decomposed into its three-color channels, and the Euclidean distance is employed [13] to determine the difference between the three distances for each color:

(9) $$\begin{array}{c} d_{i}\left(x_{\text{red}},x_{\text{blue}}\right)=\sqrt{\overset{P}{\underset{k=1}{\sum }}{\left(x_{\text{red}}^{k}-x_{\text{blue}}^{k}\right)}^{2}},\\ d_{2}\left(x_{\text{red}},x_{\text{green}}\right)=\sqrt{\overset{P}{\underset{k=1}{\sum }}{\left(x_{\text{red}}^{k}-x_{\text{green}}^{k}\right)}^{2}},\;\\ d_{3}\left(x_{\text{green}},x_{\text{blue}}\right)=\sqrt{\overset{P}{\underset{k=1}{\sum }}{\left(x_{\text{green}}^{k}-x_{\text{blue}}^{k}\right)}^{2}.} \end{array}$$

Two distances that are more alike are combined into one grayscale image and then processed as a corrected image. The proposed method is then used to determine the number of clusters to be created.

The CPSFCM consists of the following steps.

1. Divide the RGB image into three different images, use (9) to find two images that are more similar to each other, and use them to create a new grayscale image.
2. Calculate the distance between intensity levels in the image *D*, and obtain the size of an image.
3. Use this data with the fuzzy preselective system and obtain the number of centers to be created.
4. Use (8) to obtain the approximate centers. The initial value “*t*” is equal to zero, and it is used as a counter for the number of the iterations.
5. The fuzzy partition membership functions *μ*_*ij*_ are initialized according to (5).
6. Let the value be “*t* = *t* + 1” and compute the new centers using (6).
7. The steps 5 and 6 should be performed until criterion *E* converges.

## 3. Proposed Framework

In the proposed approach, the procedure consists of the following stages: a digital color image *I*[*n*, *m*]  is separated into *R*, *G*, and *B* channels in color space, where each channel image is decomposed calculating their wavelet coefficients using Mallat's pyramid algorithm [14]. Using the chosen wavelet family, the original image is decomposed into four subbands. These subbands, labeled as LH, HL, and HH, represent the finest scale wavelet coefficient (detail images), while the subband LL corresponds to coarse level coefficients (approximation image), noted below as *D*_*h*_^2^*i*^^, *D*_*v*_^2^*i*^^, *D*_*d*_^2^*i*^^, and *A* ^2^*i*^^, respectively at given scale 2^*j*^, for *j* = 1,2,…, *J*, where *J* is the number of scales used in the discrete wavelet transform (DWT). Finally, the DWT can be represented as follows:

(10) $$\begin{array}{c} W_{i}=\left|W_{i}\right|\exp\left(j\Theta_{i}\right),\\ \left|W_{i}\right|=\left(\sqrt{{\left|D_{h,i}\right|}^{2}+{\left|D_{v,i}\right|}^{2}+{\left|D_{d,i}\right|}^{2}}\right),\\ \Theta_{i}=\{\begin{array}{cc} \alpha_{i} & \text{if}\;D_{h,i}>0\\ \pi -\alpha_{i} & \text{if}\;D_{h,i}<0 \end{array}\;\;\alpha_{i}=\tan^{-1}\left(\frac{D_{v,i}}{D_{h,i}}\right). \end{array}$$

Therefore, *W*_*i*_ is considered a new image for each color channel. The following process, conducted in wavelet transform space, consists of several stages: the classic segmentation method is applied to each channel image; the segmented image corresponding to the red channel is interpolated with the segmented image corresponding to the green channel, and after applying the NNI process, the resulting image is interpolated with the segmented image corresponding to the blue channel using NNI again. Finally, this image is considered the output of the segmentation procedure.

**Figure 3:**
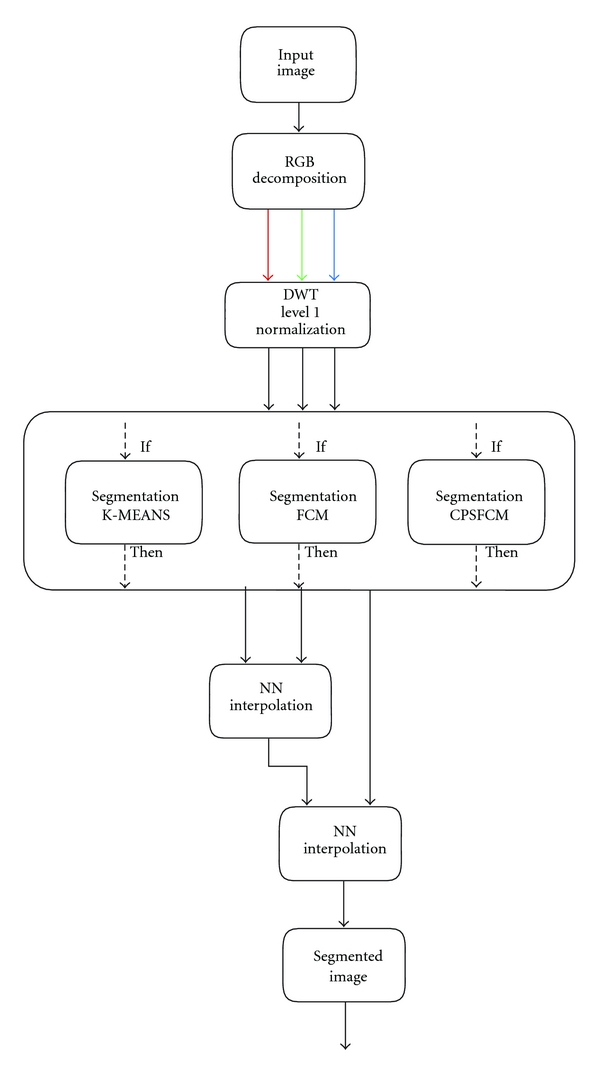
Block diagram of the proposed algorithms: WK-MEANS, W-FCM, and W-CPSFCM.

The block diagram in Figure 3 explains in detail the operations for the following: (a) image segmentation using the K-Means algorithm where WT is applied, named WK-Means, (b) image segmentation using the FCM algorithm where WT is applied, named W-FCM, and, finally, (c) image segmentation using the CPSFCM algorithm where WT is applied, named W-CPSFCM.

## 4. Evaluation Criteria

Different objective measures are used in the literature for the purpose of evaluation of the performance of border detection in dermoscopic images.

Objective measures require a ground truth (GT) image, which is determined by a dermatologist manually drawing the border around the lesion. Using a GT image, Garnavi et al. [15] calculated the operation exclusive disjunction (XOR) measure. Other metrics used in segmentation performance are presented in [16, 17] and include the *sensitivity* and *specificity*, precision and recall, *true positive rate, false positive rate*, *pixel misclassification probability*, and the *weighted performance index*.

Below, let us consider the sensitivity and specificity measure. Sensitivity and specificity are statistical measures of the performance of a binary classification test, commonly used in medical studies. In the context of segmentation of skin lesions, sensitivity measures the proportion of actual lesion pixels that are correctly identified as such. Specificity measures the proportion of background skin pixels that are correctly identified. We give the following definitions.

TP: true positive, lesion pixels correctly classified as lesion.
FP: false positive, skin pixels incorrectly identified as lesion.
TN: true negative, skin pixels correctly identified as skin.
FN: false negative, lesion pixels incorrectly identified as skin,

where, in each of the above categories, the sensitivity and specificity are given by

(11) $$\begin{array}{c} \text{sensitivity}=\frac{\text{TP}}{\text{TP}+\text{FN}},\\ \text{specificity}=\frac{\text{TN}}{\text{FP}+\text{TN}}. \end{array}$$

We also apply the receiver operating characteristic (ROC) analysis that permits us to evaluate the image segmentation quality in terms of the ability of human observers or a computer algorithm using image data to classify patients as “positive” or “negative” with respect to any particular disease. This characteristic represents the second level of diagnostic efficacy in the hierarchical model described by Fryback and Thornbury [17]. The points of the ROC curve are obtained by sweeping the classification threshold from the most positive classification value to the most negative and can be used to produce quantitative summary measures of the ROC curve for this measure called the *area under the ROC curve *(AUC).

## 5. Simulation Results

**Figure 4:**
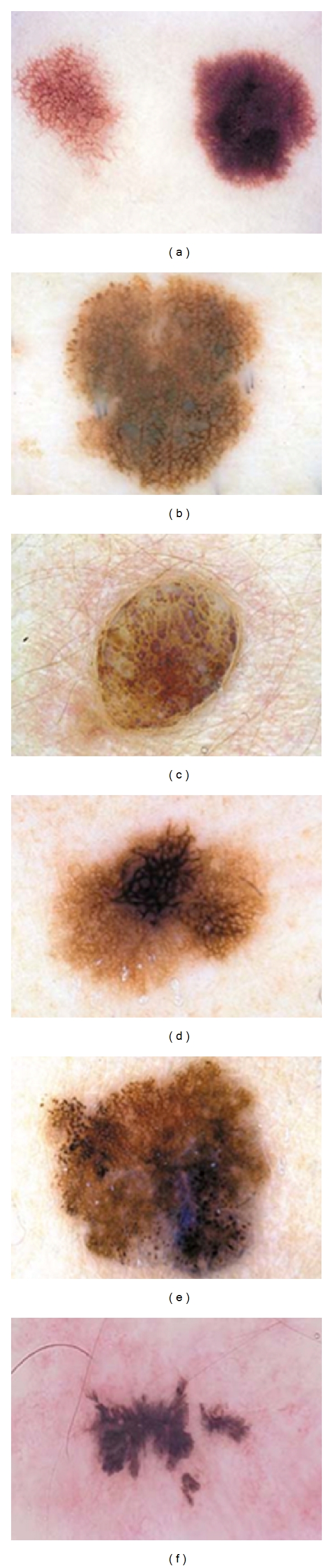
Dermoscopic images used in this study: (a)* Clark's nevus* (lesion 1), (b)* Clark nevus's* (lesion 2), (c)* dermal nevus* (lesion 3), (d)* melanoma *(lesion 4), (e)* melanoma *(lesion 5), (f)* recurrent nevus* (lesion 6).

**Figure 5:**
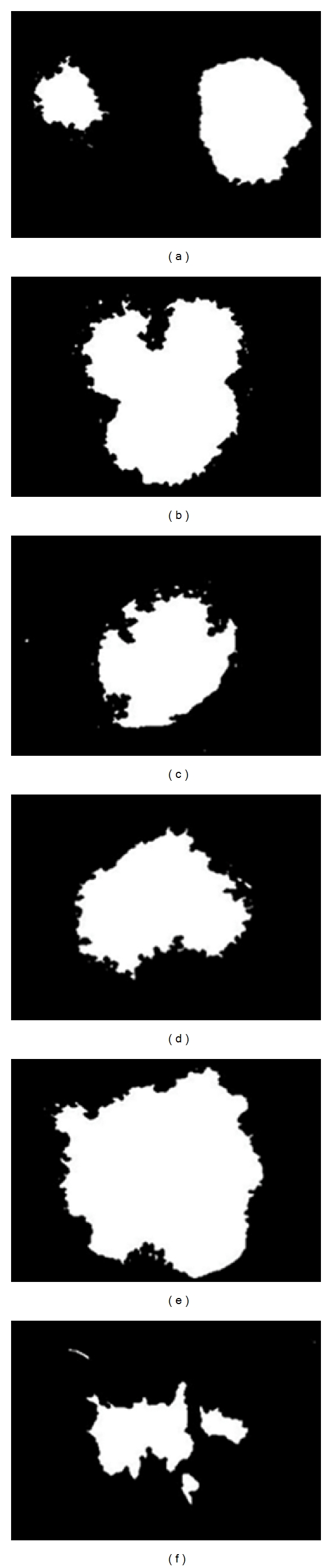
GT of dermoscopic images used in this study: (a)* Clark nevus's* (lesion 1), (b)* Clark's nevus* (lesion 2), (c)* dermal nevus* (lesion 3), (d)* melanoma *(lesion 4), (e)* melanoma *(lesion 5), (f)* recurrent nevus* (lesion 6).

The proposed segmentation algorithms were evaluated on a set of 50 images of dermoscopic images obtained from http://www.dermoscopyatlas.com [18]. These images do not contain occlusions because the preprocessing procedure has already been applied. The *GT* images were obtained via human-based segmentation (see Figure 5). The dataset presents 24-bit color images in JPEG format with 600 × 600 pixel size. Below, we expose only five different images with different texture characteristics where the *sensitivity *and *specificity* are used as the evaluation criteria for segmentation accuracy. We also plotted the ROC curves to examine the classifier performance. Additionally, the diagnostic performance was quantified by the AUC measure.  Figure 4 shows the dermoscopic images used in this study.

The simulation results in Table 4 present the AUC values for the proposed framework based on different wavelet families and confirm their improved performance compared to classical techniques. The maximum AUC value is obtained when WF Daubechies 4 is used followed by the WAF *π*_6_.

According to [16], AUC values should be greater than 0.8 to be considered a good test, but our study is focused on the best approximation of a segmented image to GT, meaning that the goal is to achieve an AUC value of approximately one.

**Figure 6:**
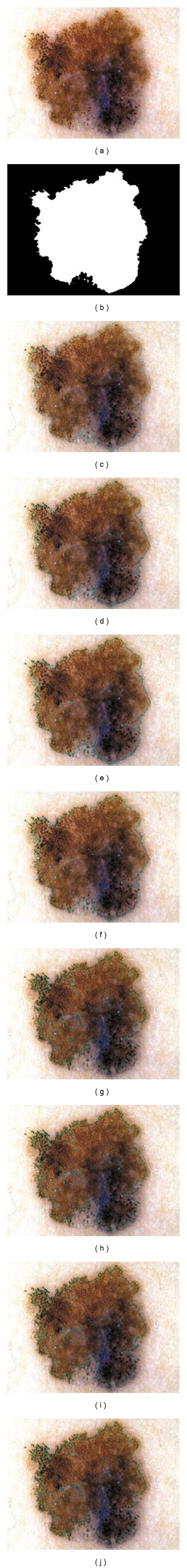
Image segmentation results under different algorithms using (a) *melanoma,* (b) *ground truth*, (c) FCM, (d) W-FCM with WF Coiflets 3, (e) W-FCM with Daubechies 4, (f) W-FCM with WF biorthogonal 6.8, (g) W-FCM with WAF up_2_, (h) W-FCM with WAF *π*_6_, and (i) W-FCM with WAF fup_2_, (j) W-FCM with WAF *e*_2_.

Figures 6(a) and 6(b) present the skin lesions and their corresponding GT. In Figures 6(c) and 6(f), it is easy to note that the segmentation procedure has selected the area only around the lesion. On the other hand, in Figures 6(g) and 6(j), where WAF results are presented, one can see that together with segmentation of the lesion border, there are some skin areas included in the lesion segment.

**Figure 7:**
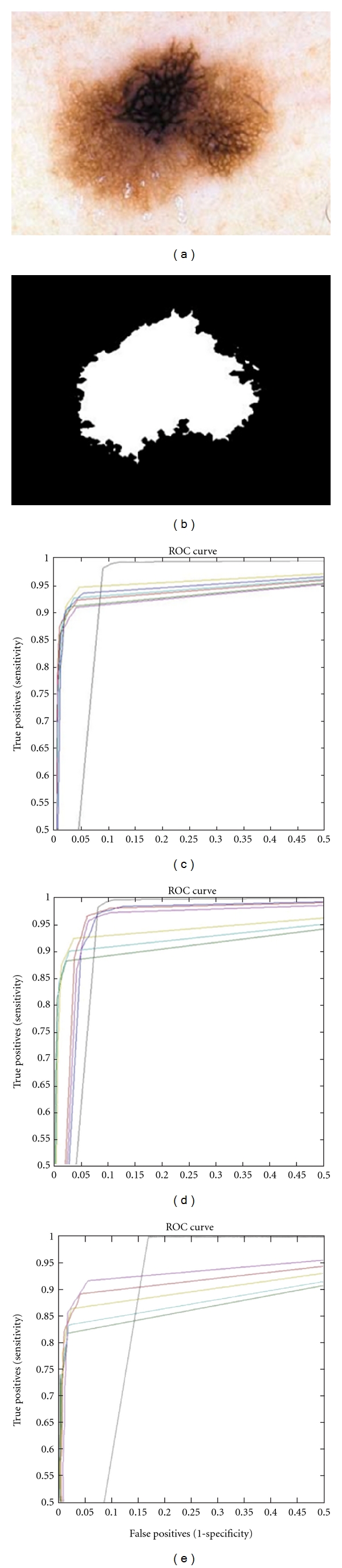
(a) Lesion 4 *melanoma,* (b) *ground truth* image; ROC curves for (c) WK-Means algorithm, (d) FCM algorithm, and (e) W-CPSFCM: for WF Daubechies 4 (dark blue), for WF biorthogonal 6.8 (red), for WF Coiflets 3 (purple), for WAF up_2_ (dark green), for WAF fup_2_ (aqua), and for WAF *π*_6_ (light green); FCM (black).

Figure 7 presents ROC curves for lesion 4 comparing the classic and proposed algorithms. In particular, Figure 7(c) presents the ROC curves for the WK-means and K-Means algorithms where one can see the superiority of the proposed WK-Means algorithm that uses WAF *π*_6_ (see ROC curve in light green color).  Figure 7(d) presents the ROC curves for the W-FCM and FCM algorithms where it is easy to observe the better performance of WK-Means that employs the WF biorthogonal 6.8 (see ROC curve in red color). Finally, in Figure 7(e), the ROC curves for the W-CPSFCM and CPSFCM algorithms have confirmed the better performance of the first one for WF biorthogonal 6.8 usage (see ROC curve in red color). The method marks one boundary around the principal lesion and sometimes other discontinues regions that can be the regions of speared lesions. All marked clusters are important for evaluation and classification processes.

## 6. Contributions of This Work

Many authors have studied the segmentation problem in dermoscopic images. The principal contribution of the current proposal resides in the use of information from all color channels together during image segmentation. We first propose an approach that involves the wavelet transform space via decomposition process in the segmentation process, employing different wavelet families. Then, the interpolation procedure between every two channels is used, finally gathering detail information of three color channels of the output segmented image. Another achievement of the proposed framework, in our opinion, is the designed pre-selective clusters system, which determines the number of clusters automatically, to analyze with the color channel images. This preselective system optimizes the FCM framework. A disadvantage of the proposed preselective system consists of an additional program intervention that may be needed for clusters with zero pixel values during channel interpolation stage. 

## 7. Conclusion

In this paper, we present three novel algorithms *W-FCM*, *W-CPSFCM,* and *WK-Means* that are applied in segmentation of dermoscopic images. All of these frameworks are compared with analogue ones that do not apply wavelet transform. The segmentation objective measures have been performed using sensibility, specificity, and AUC metric. The ROC curve analysis is also applied confirming that the usage of wavelet transform features is very promising in segmentation of dermoscopic images producing sufficiently good results. The proposed W-CPSFCM algorithm employs an additional procedure permitting to find a number of clusters in automatic mode without the intervention of a specialist. In the future, we suppose to develop the lesion classification framework using the current segmentation method.

## Figures and Tables

**Table 1:** Member functions of “Distance.”

Fuzzy set	Function	Center	Variance
*Minimum*	Gauss	15	16
Shorter	Gauss	53	24
Short	Gauss	105	30
Regular	Gauss	150	30
Large	Gauss	222	45
Maximum	Gauss	255	15

**Table 2:** Member functions of “Size.”

Fuzzy set	Function	Center	Variance
*Min*	Gauss	9000	1.789*e* + 005
Small	Gauss	3.015*e* + 005	1.626*e* + 005
Medium	Gauss	6.53*e* + 005	1.968*e* + 005
Big	Gauss	9.728*e* + 005	2.236*e* + 005
Max	Gauss	1.44*e* + 006	2.862*e* + 005

**Table 3:** Member functions of “Clusters.”

Fuzzy set	Function	Center	Variance
*Very Few*	Gauss	2	3
Few	Gauss	7	3
Some	Gauss	16	5
Many	Gauss	23	5
Too Many	Gauss	33	7

**Table 4:** AUC simulation results using different segmentation algorithms.

	Lesion 1	Lesion 2	Lesion 3	Lesion 4	Lesion 5		Lesion 1	Lesion 2	Lesion 3	Lesion 4	Lesion 5
Without wavelet						WAF Up_2 _					
CPSFCM	0.954	0.915	0.530	0.914	0.946	W-CSPFCM	0.798	0.787	0.886	0.906	0.921
FCM	0.967	0.936	0.955	0.954	0.960	W-FCM	0.826	0.929	0.901	0.935	0.913
K-Means	0.969	0.935	0.955	0.952	0.959	WK-Means	0.858	0.957	0.922	0.950	0.925
SRM	0.856	0.930	0.877	0.929	0.801						

WF Coiflets 3						WAF *π*_6_					

W-CSPFCM	0.851	0.841	0.923	0.948	0.932	W-CSPFCM	0.832	0.956	0.887	0.929	0.943
W-FCM	0.966	0.948	0.956	0.961	0.963	W-FCM	0.874	0.953	0.926	0.953	0.931
WK-Means	0.871	0.959	0.928	0.953	0.928	WK-Means	0.898	0.961	0.941	**0.965**	0.934

WF Daubechies 4						WAF fup_2_					

W-CSPFCM	0.886	0.956	**0.961**	0.958	0.961	W-CSPFCM	0.811	0.758	0.868	0.914	0.936
W-FCM	**0.969**	0.945	0.957	0.959	**0.970**	W-FCM	0.846	0.940	0.911	0.943	0.920
WK-Means	0.874	**0.964**	0.937	0.960	0.939	WK-Means	0.878	0.960	0.931	0.957	0.931

WF Biorthogonal 6.8						WAF *e*_2_					

W-CSPFCM	0.878	0.939	0.913	0.955	0.947	W-CSPFCM	0.811	0.763	0.870	0.911	0.935
W-FCM	0.966	0.949	0.956	0.962	0.964	W-FCM	0.844	0.939	0.910	0.942	0.919
WK-Means	0.869	0.958	0.927	0.953	0.928	WK-Means	0.875	0.960	0.929	0.959	0.932
